# A question of trust: can we build an evidence base to gain trust in systematic review automation technologies?

**DOI:** 10.1186/s13643-019-1062-0

**Published:** 2019-06-18

**Authors:** Annette M. O’Connor, Guy Tsafnat, James Thomas, Paul Glasziou, Stephen B. Gilbert, Brian Hutton

**Affiliations:** 1 College of Veterinary Medicine, Iowa State University, Ames, IA 50011 USA; 2 Australian Institute of Health Innovation, Macquarie University, Sydney, Australia; 3 University College London, London, WC1E 6BT UK; 4 Bond University, Robina, QLD 4226 Australia; 5 College of Engineering, Iowa State University, Ames, IA 50011 USA; 6 Knowledge Synthesis Unit, Ottawa Hospital Research Institute, Ottawa, ON K1H 8L6 Canada

**Keywords:** Artificial intelligence, Automation, Data extraction, Machine learning, Screening

## Abstract

**Background:**

Although many aspects of systematic reviews use computational tools, systematic reviewers have been reluctant to adopt machine learning tools.

**Discussion:**

We discuss that the potential reason for the slow adoption of machine learning tools into systematic reviews is multifactorial. We focus on the current absence of trust in automation and set-up challenges as major barriers to adoption. It is important that reviews produced using automation tools are considered non-inferior or superior to current practice. However, this standard will likely not be sufficient to lead to widespread adoption. As with many technologies, it is important that reviewers see “others” in the review community using automation tools. Adoption will also be slow if the automation tools are not compatible with workflows and tasks currently used to produce reviews. Many automation tools being developed for systematic reviews mimic classification problems. Therefore, the evidence that these automation tools are non-inferior or superior can be presented using methods similar to diagnostic test evaluations, i.e., precision and recall compared to a human reviewer. However, the assessment of automation tools does present unique challenges for investigators and systematic reviewers, including the need to clarify which metrics are of interest to the systematic review community and the unique documentation challenges for reproducible software experiments.

**Conclusion:**

We discuss adoption barriers with the goal of providing tool developers with guidance as to how to design and report such evaluations and for end users to assess their validity. Further, we discuss approaches to formatting and announcing publicly available datasets suitable for assessment of automation technologies and tools. Making these resources available will increase trust that tools are non-inferior or superior to current practice. Finally, we identify that, even with evidence that automation tools are non-inferior or superior to current practice, substantial set-up challenges remain for main stream integration of automation into the systematic review process.

## Background

Systematic reviews are a critical component of evidence-informed policy making in clinical health, public health, software engineering, environmental policy, food security and safety, and business management [1–9]. Current approaches to the conduct of systematic reviews, typically taking months or years [10], are a rate-limiting step in the rapid transfer of information from primary research to reviews, because the process is slow and human-resource intensive. Further, with increasing movement toward living reviews, such efforts will require technological advances in order to be sustainable [11, 12]. One solution to reducing the workload of systematic reviews is to incorporate automation technology software including algorithms that operate on information, and tools that allow users to invoke such algorithms.

Different domains involving automation, such as autonomous vehicles, use different frameworks to describe the varying levels of automation (see Vagia et al. [13] for a review of levels of automation frameworks). One such framework, which we apply to systematic reviews, is provided in Table 1. Although automation tools capable of Level 3 and Level 4 tasks are rapidly becoming available for systematic reviewers, surveys suggest that adoption of these automated technologies by the systematic review community is slow [7].

**Table 1:** Levels of automation for human-computer interactions

Level	Task
Level 4	Tools perform tasks to eliminate the need for human participation in the task altogether, e.g., fully automated article screening decision about relevance made by the automated system.
Level 3	Tools perform a task automatically but unreliably and require human supervision or else provide the option to manually override the tools’ decisions, e.g., duplicate detection algorithms and software, linked publication detection with plagiarism algorithms and software.
Level 2	Tools enable workflow prioritization, e.g., prioritization of relevant abstracts; however, this does not reduce the work time for reviewers on the task but does allow for compression of the calendar time of the entire process.
Level 1	Tools improve the file management process, e.g., citation databases, reference management software, and systematic review management software.

Currently, few systematic review teams use automation technology to take over all, or some, of the cognitive tasks or to take on a higher level of decision-making or interpretation (Levels 3 and 4) [14]. For example, despite numerous studies that are over a decade old documenting the use of machine learning approaches to screening citation records, this technology remains rarely used in peer-reviewed systematic reviews [15–20]. Further, when machine-assisted screening is used, the approach is usually limited to Level 2 automation. In the most common approach to using machine-assisted screening, after training on a subset of studies classified as relevant or not by the human reviewer, the automation tool reorders the citations from highest to lowest probability of being relevant to the review. The human reviewer is still required to make the final decision on all citations. This approach to screening does compress the time required to conduct the entire review but does not dramatically reduce the time on task of an individual reviewer. Similarly, although tools exist for detecting duplicate publications, many teams require a reviewer to verify duplicates before excluding studies. Therefore, transitioning to Level 3 and 4 automation, where the automation tool independently makes some decisions, is critical if the real resource savings of automation are to be realized.

## Barriers to adoption of automation

Given this absence of adoption of automation technologies by systematic reviewers, it is of interest to understand what the potential barriers are. We hypothesize that these barriers include (a) mistrust by the reviewer team or end users in the automation tools, (b) set-up challenges, e.g., matching tools with data formats and/or integration of tools into current review production processes, (c) the ability of the automation technology to perform the task, and (d) awareness of available tools, i.e., making people aware of what is available. While all these barriers to adoption are critical, the focus of this manuscript is on the first two items: issues of trust and the role that set-up challenges might play in slow adoption.

There are several theories associated with the adoption of technology [21, 22]. Here we focus on the *diffusion of innovations* theory which proposes that the rate of innovation adoption is affected by five characteristics:

- Characteristic 1. Being perceived as having a greater relative advantage,
- Characteristic 2. Compatibility with current practice,
- Characteristic 3. It is possible to “trial” the new technology,
- Characteristic 4. Observing others doing the same, and
- Characteristic 5. Reduction of complexity [22].

Of these issues, we hypothesize that the issue of *compatibility with current practice* (Characteristic 2) is most closely related to our concept of trust and set-up challenges [23]. C*ompatibility with current practice* can have two dimensions. Firstly, compatibility with current practice as it relates to the product delivered. To illustrate this concept, we use electric cars as an analogy. For electric cars, a compatible product would be an electric car able to drive 100 km per hour for 300 km on a single charge, which would be a reasonable expectation for a modern car on a single tank of gasoline. For a systematic review this would mean a review developed using automation is equivalent or superior to the current practice.

Secondly, compatibility with current practice as it relates to the process used to develop the product. Using the electric car analogy again to illustrate this idea, a compatible process would have the ability to utilize the same factories and staff to produce the electric car as used to manufacture gasoline cars. For a systematic review, this would mean that any changes required can seamlessly be integrated into the current resources including software currently used without major disruption or relearning of processes required.

### Compatibility with current practice—building trust

With respect to the outcome, certainly within clinical and public health, systematic reviews are recognized as a trusted product used to develop policy. This trust has been built over many years and although many policy makers are perhaps unaware of how reviews are actually produced, they trust the product. However, trust that the current system produces high-quality reviews is also likely to result in concern that an approach that deviates from the current system might not be of equal quality. Therefore, tools at the Level 3 and Level 4 automation levels must not be perceived as an erosion of current practice standards. This compatibility issue could be addressed if automated methods were trusted and known to be valid.

Based on the *diffusion of innovations* theory, we propose that in the areas of clinical and public health, where the current standard approach is well established and highly regarded, increasing adoption of automated tools that involve some level of decision-making (Levels 3 and 4) will require credible evidence that the automation tool is non-inferior or superior in accuracy to current practice, i.e., documented greater relative advantage. Further, review teams and end users, such as funding agencies, guideline developers, and policy makers must be persuaded that “others” also consider the reviews produced using new approaches as non-inferior or superior in accuracy to current practice. This latter issue is particularly problematic and also incorporates Characteristic 4 of the *diffusion of innovations* theory, observing others doing the same.

If a review team thinks there is a risk of rejection of a grant application or an article because a grant panel, peer reviewer, or editors considered the methods incompatible with current practice, then the benefit of reduced completion time at reduced cost will not be sufficient to cover the negative impact of grant or publication rejection. Even if an automation tool has been shown to make identical (or better) decisions to the human reviewer while also being cheaper and faster, in this scenario the automation technology will not be used, as the harms outweigh the benefits. This means the review community needs two factors before widespread adoption can realistically occur. Clearly, there is a need for studies focused on documenting non-inferiority or superiority in accuracy of automated approaches compared to current practice. But of equivalent importance, some highly regarded review teams, groups overseeing reviews, or funding agencies need to take the lead in funding or producing reviews that use automation tools. These highly credible early adopters will serve as empirical documentation that automated approaches are trusted and pave the way for a critical mass of review teams to also adopt the tools.

### Compatibility with current practice—set-up challenges

#### Current culture of work tasks can be a barrier to adoption of tools

As mentioned above, the *compatibility with current practice* (Characteristic 2) in the *diffusion of innovations* theory can have two dimensions: compatibility with current practice as it relates to the product delivered (discussed above) and compatibility with current practice as it relates to the process used to develop the product. Our electric car analogy described a compatible process as being able to utilize the same factories and staff to produce the electric car as are used to manufacture gasoline cars. For a systematic review, this would mean that any automation technologies required can seamlessly be set up with minimal disruption to processes and resources including software, staffing, and staff skills. We anticipate that the current culture of systematic reviews, in some areas and groups, contributes to the set-up challenges and these barriers will exist even if highly regarded teams or funding agencies lead the way by using automation tools to produce reviews.

Despite the fact that many automation tools exist (at the time of writing 159 software tools are indexed on the systematic review toolbox website- http://systematicreviewtools.com) and more being developed monthly, it is unclear how many can seamlessly be set up in each unique review team workflow. Therefore, another barrier to adoption is the combined effect of inertia to adoption associated with a “known process” and the difficulties associated with integrating automated tools into that “known process.” Although the systematic review process on paper is described as a linear process of tasks and subtasks [24–28], the management and variety of the process can be quite complex. Here we differentiate the flow of work, which refers to the order in which tasks occur, from the work task approach which is how the tasks (subtasks) are done.

Knight et al. [14] recently provided a fascinating insight into the actual workflow and work tasks of a single systematic review group, the Cochrane Schizophrenia Group (CSzG) at the Institute of Mental Health, University of Nottingham. The description of the process highlighted how “institutional” or “local” the actual approach used to conduct the systematic review process can be for different teams. For example, the CSzG stated “The data are simply extracted onto sheets of paper (Figure ..) and then entered later into the review writing software”. While this would be recognizable as the data extraction process of some review teams, many review teams do not use this paper to software process, and so an approach designed to automate this work task may not be useable or relevant for other teams.

Similarly, Knight et al. [14] described that “A vital part of all strategies for data extraction is the annotation of the source documents to indicate the location of the evidence for the data in the forms. This annotation may take the form of highlighting sentences or phrases (see Figure ..), or placing small numbered marks in the forms that are then referred back to.” However, it is not the case that all teams incorporate this annotation task or numerical tagging approach into data extraction. Even within teams, Knight et al. [14] described different approaches used by novice versus expert reviewers even for a single task such as data extraction (see Figure 3 of [14]). These examples from Knight et al. [14] show that when software developers create a tool to replace a step in the systematic review process such as data extraction, the work tasks being replaced may actually differ between review teams. For example, a developer working with the CSzG group might incorporate PDF file annotation into data extraction. When a different team attempted to adopt that tool, it would not seamlessly fit into the set of tasks already in place, and might actually add a task. This additional task might be a barrier to adoption. These differences in process mean that making tools compatible with current practice can be difficult and often not generalizable. It will require a change in culture and work practices even for validated tools to be adopted. Done in conjunction with a review team, adoption and integration into a workflow may not transfer as expected because although the step of the review is the same, i.e., data extraction, the work tasks might be different.

#### Automation may facilitate (or require) disruption of the current workflow

Another related challenge for automation, even of trusted tools, is that it might require disruption of the current workflow, which could require redistribution of work duties and new skills acquisition for staff. Currently, the workflow of systematic reviews is described as linear and the number of tasks and subtasks differs between authors. Regardless, the approach generally implies the steps are completed in a particular order. For example, for any particular citation, it is retrieved and screened for relevance, the full text is retrieved and screened for relevance again, data are extracted, and risk of bias is assessed. Eventually, all citations must “meet” at the same point for synthesis and summarization. This process currently implies a system of staff responsibilities and skills. However, it is possible to envision that automation might not need such a workflow. For example, a review group might use automated approaches to extract all characteristics and results data from all studies about a certain topic as soon as published, and simply store these data for later retrieval when a review is requested. This approach clearly puts data extraction even before review question development and protocol development, and such an approach would enormously disrupt current workflow. Because of the inertia to change that occurs in many groups, this would be a barrier to adoption.

### Designing automation assessment studies

Some of the barriers to adoption we have discussed require cultural change and how to effect that change is beyond the scope of this manuscript. However, it is obvious that first and foremost, there must be evidence that automation tools produce non-inferior results from primary studies.

With respect to designing studies that document non-inferiority or superiority in accuracy of automated approaches compared to current practice, many automation tasks such as screening citations, screening full texts, risk-of-bias assessment, and data extraction can be framed as accuracy evaluations, similar to the assessment of diagnostic tests. For example, for screening abstracts, the desired information is “Do the human and the algorithm both categorize (classify) a citation as being relevant to the review?” Similarly, for automated risk-of-bias assessment, the desired information is, “Do the human and the algorithm both categorize (classify) a study as being at high, unclear, or low risk of bias?” Both of these can fairly easily be understood as variations of diagnostic test evaluation [29, 30].

Data extraction can also be considered a classification experiment [31, 32]. The goal of this task is to extract text about characteristics of the study described in the manuscript (e.g., a clinical trial). Groups of words in the text are classified as being descriptive of the characteristic of interest or not. As clinicians and public health officials are very comfortable with diagnostic test evaluations and the metrics used to assess these tools, we see this as an opportunity to leverage this comfort to build trust in automated tools. Conceptually, classification experiments are relatively simple to design. A single population which contains a mixture of classes is categorized using all available tools. Ideally, a gold standard classification is available.

### Outcome metrics for automation assessment studies

The standard metrics for comparison of classification methods should be employed as appropriate for the classification problem: average precision, recall, and F_1_ scores in information retrieval tasks, sensitivity, specificity, and area under the receiver operating characteristic curve (The latter is often abbreviated as AUC, ROC, or sometimes AUROC.) in classification tasks and strict vs. relaxed variants in natural language processing (NLP) tasks: these and other summary measures of relevance have been described elsewhere [17].

If the classification is not binary, these metrics can be extended to multi-classification problems. It is possible that these estimations could be obtained by assuming at least one classifier, usually the human as a gold standard, and using cross-validation for supervised classification tasks. Alternatively, it might be valid to assume that non-perfect measurement of both classifiers exists and for the performance metrics to be obtained using latent class methods of determination of sensitivity and specificity [33, 34].

As the time saved is also part of the greater relative advantage equation of adoption (Characteristic 1 of the *diffusion of innovations* theory), additional metrics that reflect time saved by using the classifier are also likely of interest. Currently, the most common examples are the percentage and number of citations screened to detect all relevant studies, or the percentage of relevant citations identified by a set threshold (50%, 80%, 95%).

### Reporting of automation assessment studies

In clinical practice, the standards for reporting diagnostic test evaluations are well established, and adherence allows for assessment of bias [35]. However, reporting software experiments that include comparison of human reviewers to software algorithms or comparison of multiple software algorithms and a corpus of papers presents new challenges for reproducible reporting. In a recent publication reviewing the automated citation screening methods reported for systematic reviews, the number of studies that met the current standards for reproducible software engineering experiments was low [29, 30, 36]; of 33 studies reporting approaches to automated screening, no study provided all the criteria for reproducible reporting [36]. This poor reporting might be related to the lack of development of trust.

It seems likely that the systematic review community, which often focuses on, and is often critical of, the quality of primary research, will find it challenging to trust technologies where the primary evaluation research falls below their acceptable standards. As a consequence, in order to build trust, authors of reports about automation should adhere to the standards available for reliably reporting software experiments [36]. The proposed checklist by Olorisade et al. [36] should be a critical guide for reporting all proposals and reports.

### Sharing data from automation assessment studies

Classification experiments are usually considered more valid (trustworthy) if an acceptable gold standard is used for the classification. However, developing a gold standard corpus of papers for each classification target requires considerable investment in human time. It is potentially wasteful for each algorithm developer to also develop a new evaluation corpus. To rapidly improve the pace of research in this area, it would be ideal if software developers had access to high-quality, validated datasets, for real systematic review tasks. The availability of validated datasets that could be used by developers to train and evaluate automation approaches will raise the quality of evaluation of automation tools by serving as benchmarks.

Datasets should comply with current and evolving standards for public datasets and corpora [29, 30, 36, 37]. For classification experiments, we envision two possible formats for presenting such datasets: (1) as a spreadsheet of classification results with the supporting text or as a corpus of annotated files (or processed texts) providing classifications and supporting text. For data shared as spreadsheets, in addition to the normal standards for reporting the corpus, investigators should provide the metadata about the classification task(s). This information would explain the classification task(s) assessed, the possible values, and instructions on how to interpret each annotation. Table 2 provides an example of additional data relevant to systematic reviews that might be included in publicly shared data.

**Table 2:** Proposed additional items for inclusion in a shared dataset for a classification experiment for automation of systematic review processes

Column	Item
1	Title of source—publication name, report name, etc.
2	Indexing data (e.g., PubMed identifier, ISBN, doi)
3	Author names
4	Publication venue (e.g., journal name)
5	Serial data (e.g., volume, issue, and page numbers)
6	A final classification field. This would be a final category used in the systematic review. For example, if the dataset is designed for screening, this field might refer to inclusion status in the final systematic review (“yes” or “no”), or if the classification task is bias assessment this might refer to bias assessment in the final systematic review (“low”, “high”, “unclear”).
7	Reviewer 1 classification, i.e., whether Reviewer 1 recommended inclusion of the article in the systematic review
	Reviewer 1 notes field (free text) whenever notes were provided by the reviewer
8	Reviewer 1 notes field supporting text from the manuscript if extracted (optional)
9	Reviewer 2 classification, i.e., whether Reviewer 2 recommended inclusion of the article in the systematic review
10	Reviewer 2 notes field (free text) whenever notes were provided by the reviewer
	Reviewer 2 notes field supporting text from the manuscript if extracted (optional)
11	Arbiter notes field (free text) whenever notes were provided by the arbiter
12	A training field (“yes” or “no”) on whether the entry was used to train human reviewers

For datasets shared as annotated research report files, Table 3 provides examples of approaches to archiving. The metadata are provided separately from the corpus, and descriptions of the annotation process are included in the metadata. The rules for identifying supporting text should be provided, i.e., phrases, complete sentences, and text between punctuation marks should be included. Incorporation of a mechanism for the community to correct errors that may exist in the original dataset would be ideal. However, given that not all groups have continued funding for such efforts, we would not consider this a requirement for shared datasets as that may limit enthusiasm for sharing.

**Table 3:**
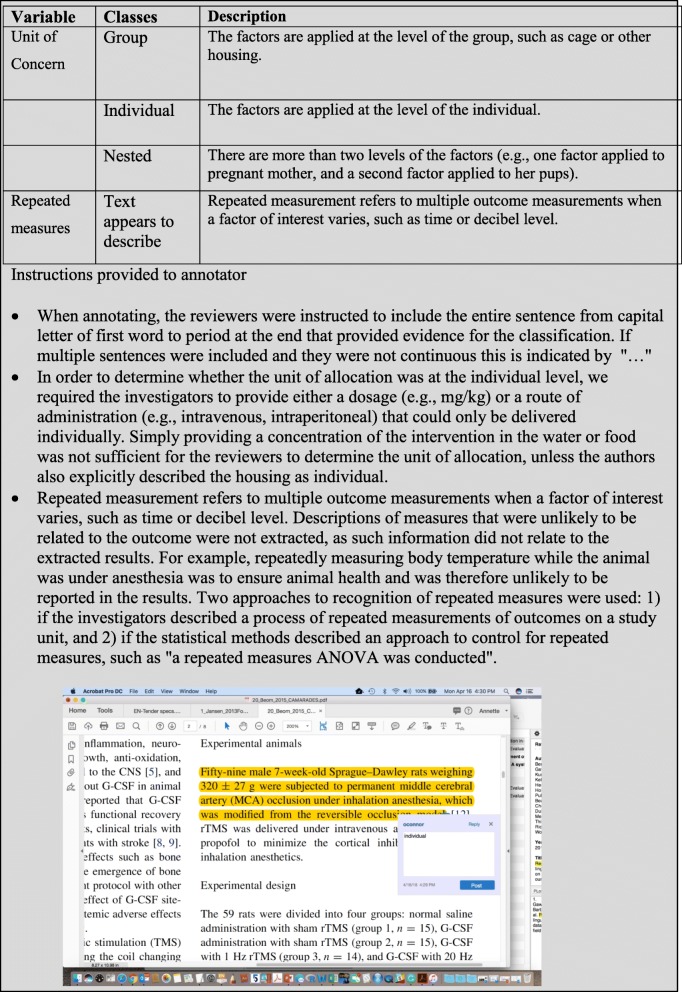
Illustration of the proposed additional metadata documentation for sharing files annotated for systematic reviews

## Conclusion

Automation of systematic reviews has the potential to increase the speed of translation of research to policy, reduce research wastage, and improve health outcomes. However, there are many technological and adoption barriers to using automation methods in systematic reviews. Our focus here was on adoption barriers, and we proposed that lack of trust and set-up challenges are key causes for reluctance to adopt automation tools. To build trust, the systematic review community needs studies that build a trusted evidence base and leadership from early adopters. Such an evidence base consists of classification experiments that address the accuracy of classification and comparative assessments of work time. Although the designs for these studies are well-known, in software experimentation, unique challenges arise that should be addressed before studies are conducted and reported. Even with validated tools used by highly regarded teams, adoption of automation technologies by a critical mass of review teams faces challenges because integration of the automation technology into the workflow and work tasks remains a barrier.
